# Evaluation of the AGE/sRAGE Axis in Patients with Multiple Myeloma

**DOI:** 10.3390/antiox8030055

**Published:** 2019-03-04

**Authors:** Alessandro Allegra, Caterina Musolino, Elisabetta Pace, Vanessa Innao, Eleonora Di Salvo, Maria Ferraro, Marco Casciaro, Giovanna Spatari, Gennaro Tartarisco, Andrea Gaetano Allegra, Sebastiano Gangemi

**Affiliations:** 1 Division of Hematology, Department of Human Pathology in Adulthood and Childhood “Gaetano Barresi”, University of Messina, 98125 Messina, Italy; cmusolino@unime.it (C.M.); vinnao@unime.it (V.I.); andrea.allegra@hotmail.it (A.G.A.); 2 Institute of Biomedicine and Molecular Immunology “A. Monroy” (IBIM), National Research Council (CNR), 90146 Palermo, Italy; elisabetta.pace@ibim.cnr.it (E.P.); ferraro@ibim.cnr.it (M.F.); 3 National Research Council of Italy (CNR)-Institute of Applied Science and Intelligent System (ISASI), 98164 Messina, Italy; eleonora.disalvo6@gmail.com (E.D.S.); gennaro.tartarisco@gmail.com (G.T.); 4 School and Operative Unit of Allergy and Clinical Immunology, Department of Clinical and Experimental Medicine, University of Messina, 98125 Messina, Italy; mcasciaro@unime.it (M.C.); gangemis@unime.it (S.G.); 5 Department of Biomedical Sciences, Dental, Morphological and Functional Investigations, University of Messina, 98125 Messina, Italy; gspatari@unime.it

**Keywords:** advanced glycation end products, soluble receptor of advanced glycation end products, oxidation, multiple myeloma

## Abstract

Glycative stress influences tumor progression. The aim of the present study was to evaluate the advanced glycation end products/soluble receptor of advanced glycation end products (AGE/sRAGE) axis in patients with multiple myeloma (MM). Blood samples were taken from 19 patients affected by MM and from 16 sex-matched and age-matched healthy subjects. AGE and sRAGE axis were dosed in patients with MM and matched with controls. AGEs were measured by spectrofluorimetric methods. Blood samples for the determination of sRAGE were analyzed by ELISA. AGE levels were significantly reduced in patients with respect to controls. Instead, sRAGE was significantly elevated in patients affected by MM compared to healthy subjects. Moreover, we showed that there was a statistically significant difference in sRAGE according to the heavy and light chain. IgA lambda had significantly higher sRAGE values than IgA kappa, IgG kappa, and IgG Lambda MM patients. From our data emerges the role of the sRAGE/AGE axis in MM. Since AGE is a positive regulator of the activity of RAGE, circulating sRAGE concentrations may reflect RAGE expression and may be raised in parallel with serum AGE concentrations as a counter-system against AGE-caused tissue damage. Serum concentrations of AGE and sRAGE could therefore become potential therapeutic targets.

## 1. Introduction

Glycation is a common reaction that happens in most cells between a carbonyl group (–C=O) of the reducing sugars and the amino group (–NH2) of proteins, initially leading to the creation of an unstable substance known as Schiff’s base. Schiff’s bases are short-lived and susceptible to a reverse reaction that causes the reconstruction of original reactants. They have a predisposition to undertake re-arrangement of atoms causing the development of Amadori products [1,2]. These are more durable than Schiff’s base and more prone toward the forward reaction. Furthermore, they undergo cyclization and dehydration to produce advanced glycation end products (AGEs), although AGEs can also be produced during the oxidation of lipids or nucleotides. AGEs are responsible for the onset and the evolution of several diseases, including obesity, nephropathy, diabetes, retinopathy, neuropathy, aging, and cardiovascular diseases [3].

Moreover, glycative stress caused by AGEs can considerably influence tumor progression. In fact, oxidative stress can stimulate several transcription factors, such as protein-53, activating protein-1, nuclear factor erythroid-2-related factor, hypoxia-inducible factor-1α, β-catenin/Wnt, and peroxisome proliferator-activated receptor-γ (PPAR-γ). Triggered transcription factors can conduct to about 500 diverse modifications in gene expression and can modify expression patterns of growth factors, regulatory cell cycle substances, and inflammatory and anti-inflammatory molecules. These changes of gene expression can stimulate a normal cell to become cancerous [4]. For instance, plasma concentrations of AGEs were augmented in subjects with breast cancer with respect to healthy subjects [5].

The most investigated AGE receptor is the multiligand receptor of the immunoglobulin super-family, coded in the Class III region of the major histocompatibility complex, known as the receptor of AGE (RAGE). Several in-vivo and in-vitro investigations have been centered on the cancer-promoting actions of the AGE–RAGE relationship, believed to be a decisive link between tumors and chronic inflammation. Several studies have evaluated the specific action of RAGE in controlling pro-tumorigenic pathways in immune and cancer cells, and in the microenvironment of a tumor [6,7,8], and stimulation of RAGE by AGEs was shown to increase growth or migration of melanoma and pancreatic cancer cells [9]. RAGE was upregulated in colorectal, esophageal, and oral squamous cell carcinoma. In these conditions, it acted as an oncoprotein [10].

RAGE is present at low concentrations in a broad range of adult cells, while it is extremely expressed in embryonic cells [11]. RAGE is produced as both full-length, membrane-bound forms (fl-RAGE or mRAGE) and as several soluble products missing the transmembrane domain. Soluble RAGE (sRAGE) is generated by both proteolytic cleavage of fl-RAGE and alternative mRNA splicing [12,13,14]. In recent years, RAGE control has been evaluated in several human carcinomas. In breast cancer, it was recognized that subjects with better outcomes had higher sRAGE concentrations [15]. Finally, several studies have evaluated the action of glycative stress on the onset of various onco-hematologic diseases [16,17]. 

Multiple myeloma (MM) is an incurable disease provoked by the malignant growth of bone marrow plasma cells, whose pathogenesis is fundamentally unknown. However, it is a hematological tumor with underlying causes connected to augmented oxidative stress. In a previous study, we evaluated the plasma concentrations of advanced oxidation protein products (AOPPs) and protein nitrosylation in order to measure the oxidative stress in untreated MM subjects and in subjects affected by monoclonal gammopathy of uncertain significance (MGUS) [18]. We demonstrated that plasma levels of AOPPs and S-nitrosylated proteins were considerably augmented in MM subject with respect to controls and to MGUS subjects. Moreover, we also studied serum concentrations of carbonyl groups, and these were notably higher in MM subjects as compared with healthy subjects, in particular in the more advanced stages of MM [19]. A recent study has shown that the concentrations of AGEs and RAGE are augmented in the saliva of MM subjects with bone lesions [20]. To the best of our knowledge, there are no works that have explored the soluble concentrations of RAGE in patients with MM. The aim of the present study was to evaluate the AGE/sRAGE axis in patients with MM compared to a healthy control group. We have also tried to detect the existence of correlations between this axis and the variables commonly used to evaluate MM. 

## 2. Materials and Methods

### 2.1. Patients and Control Subjects

The study was conducted at the Division of Hematology of the University Hospital of Messina “Policlinico G. Martino”, Italy. 

The study was conducted according to good clinical and laboratory practice rules and according to the principles of the Declaration of Helsinki. Informed written consent was obtained after potential risks were explained to the subjects. The study was approved by the local ethics committee (Protocol No. 36/18 of 07/05/2018-resolution No. 887).

Blood samples were taken from 19 patients affected by MM (7 males and 12 females; mean age 71.63 ± 9.96) and from 16 sex-matched and age-matched healthy subjects (Table S1). The paraprotein class was immunoglobulin G (IgG) in 14 patients and A (IgA) in 5 patients (light chain k in 12 patients, lambda in 7 patients).

None of the patients were receiving chemotherapy or anti-inflammatory drugs. None of the patients or controls had symptoms of active infections, inflammatory diseases, diabetes, obesity, or hypertension. Multiple myeloma patients were characterized using the Durie Salmon system (3 patients were in stage I, 7 stage II, and 9 stage III), and the International Staging System (ISS) (7 patients were in stage I, 7 stage II, 5 stage III). All but one patient had lytic lesions.

Routine laboratory studies consisted of complete blood count with differential, platelet count, and blood chemistry including beta-2 microglobulin, renal and liver function tests, calcemia, serum albumin, and lactate dehydrogenase (LDH), as well as immunoglobulin, erythrocyte sedimentation rate (ESR), and bone marrow aspiration. Physical examinations and skeletal X-rays were performed at all times. Blood samples were immediately centrifuged at 14,000× *g* rpm for twenty minutes and aliquoted into 1.5 mL centrifugation tubes. Samples were stored at −20 °C until tested. 

### 2.2. AGEs Analysis

We decided to assess the fluorescent AGE by the spectofluorimetric method because fluorescent AGEs (fAGEs) are considered a subset remarkably representative of the entire AGE family and fAGEs can be easily assessed in sera [21,22]. Serum was diluted 1:5 with phosphate-buffered saline (PBS: pH 7.4), and fluorescence intensity was recorded at maximum emission (440 nm) upon excitation at 350 nm and expressed in arbitrary units (AU). The serum concentration of AGEs was normalized to the total protein amount determined by the Bradford assay and expressed in AU for protein gram. Each sample was analyzed in duplicate for determination (Tables S2 and S3).

### 2.3. sRAGE Analysis

After collection of blood samples, we separated the serum and assessed sRAGE in serum by ELISA (enzyme-linked immunosorbent assay) sRAGE (R&D System human RAGE Quantikine ELISA Kit DRG00, Minneapolis, MN, USA) according to the manufacturer’s instruction. ELISA assay was run in duplicate.

### 2.4. Statistical Analysis

Data were analyzed using SPSS (version 20.0, IBM corp., New York, NY, U.S.A.) and stated as mean ± standard deviation or as median and interquartile range [IQR 0.75–0.25] when the variables were not-normally distributed. A *p* value of less than 0.05 was considered statistically significant. The difference between experimental conditions was evaluated by the Wilcoxon non-parametric test. The comparison of different types of heavy and light chain with sRAGE was performed using the non-parametric Kruskal Wallis test. When the results of the Kruskal-Wallis test were statistically significant, a comparison of groups was achieved using the non-parametric Wilcoxon rank-sum test.

Multiple linear regression analysis was used to develop a model for predicting sRAGE and AGE values from MM parameters with the highest correlation.

## 3. Results

**Figure 1:**
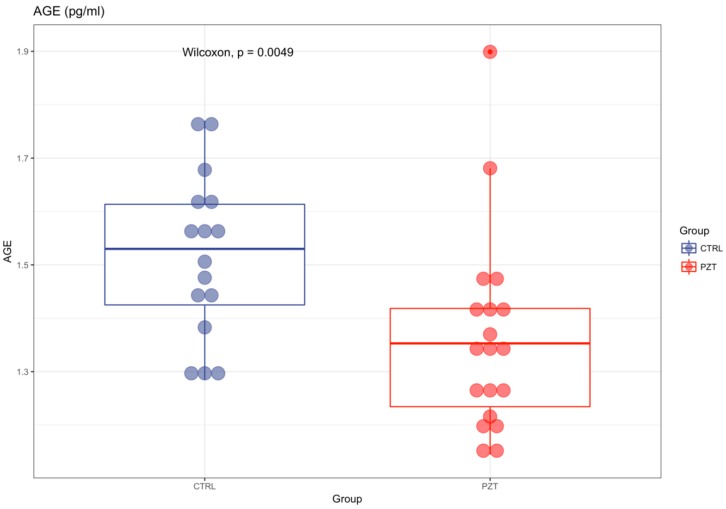
AGE levels were significantly reduced in patients with respect to controls (1.40 ± 0.2 vs 1.52 ± 0.15; *p* < 0.01).

**Figure 2:**
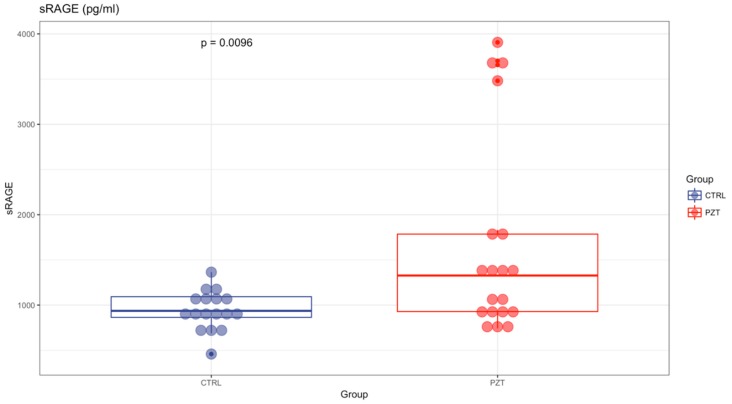
sRAGE was significantly elevated in patients affected by multiple myeloma (MM) compared to healthy subjects (1686.3 pg/mL ± 1107 vs 940.67 pg/mL ± 218; *p* < 0.01).

AGE levels were significantly reduced in patients with respect to controls (1.40 ± 0.2 vs 1.52 ± 0.15; *p* < 0.01) (Figure 1). Instead, sRAGE was significantly elevated in patients affected by MM compared to healthy subjects (1686.3 pg/mL ± 1107 vs 940.67 pg/mL ± 218; *p* < 0.01) (Figure 2). For both variables, there was no correlation with age (Table 1), and no statistical differences were evidenced for sex (*p* > 0.05). 

**Figure 3:**
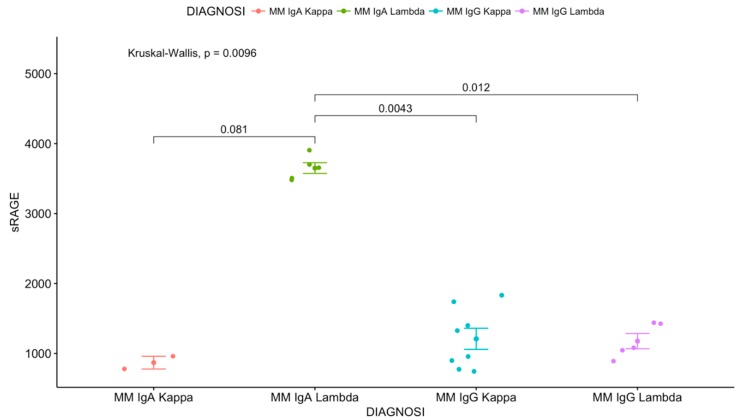
The Kruskal-Wallis H test showed that there was a statistically significant difference in sRAGE according to the heavy and light chain.

The Kruskal-Wallis H test showed that there was a statistically significant difference in sRAGE according to the heavy and light chain (Figure 3): X2(3) = 11.434, *p* = 0.0096. IgA lambda had significantly higher sRAGE values than IgA kappa, IgG kappa, and IgG Lambda (Wilcoxon rank-sum analysis: *p* = 0.081, *p* = 0.0043, *p* = 0.012, respectively).

The parameters with significant correlations with sRAGE were bone marrow plasma cells (*r* = 0.50 *p* < 0.05) and calcemia (*r* = 0.61 *p* < 0.001). We also combined other parameters such as Beta2 Microglobulin. Finally, the variables Beta 2 Microglobulin, bone marrow plasma cells, and the interaction between calcemia and bone marrow plasma cells were the best combination of model variables which significantly predicted sRAGE, F(4,15) = 14.96, *p* < 0.0005, R^2^ = 0.746 (Table 2).

We also tried to build another multiple linear regression model for AGE values prediction from MM parameters, but the best combination of model variables which significantly predicted AGE was F(2,17) = 8,52, *p* < 0.005, with R^2^ = 0.442.

## 4. Discussion

The action of the RAGE ligand(s)/RAGE system seems to be implicated in several tumors promoting the growth of tumor cells, metastasis, and resistance to therapy.

sRAGE was shown to reduce motility and pro-metastatic stimulation of A-375 melanoma cells by stopping S100A4-RAGE signaling axis [23]. Moreover, in a study of Finnish male smokers, serum concentrations of sRAGE were found inversely correlated with pancreatic cancer risk [24].

Khorramdelazad et al. demonstrated that mRNA expression of RAGE was significantly increased in bladder cancer tissue, and mRNA expression of RAGE and its receptors might also be a helpful marker for transitional cell carcinoma (TCC) [25].

Concerning AGEs and carcinogenesis, variations between intra and extracellular AGEs concentrations could, by a protein crosslink, modify cell structure and function and also augment the levels of oxidative stress through ROS (Reactive oxygen species) generation [26]. A changed mitochondrial protein function, via the blocking of the electron transport chain, increases inflammatory protein expression through mitochondrial pathway initiated apoptosis or cancer [27]. The ROS produced as result of the Maillard reaction may also provoke damage to the DNA. It is the amount of the ROS produced matters the most, as the same ROS may provoke apoptosis and may be a reason for the cancer.

Moreover, the induced cytokine production (IL-1, TNF) increases the generation of cell adhesion molecules (endothelin-1, VCAM-1, ICAM-1), vascular endothelial growth factor, and decreases the production of endothelial nitric oxide synthase [28]. Dicarbonyls/AGEs further augment inflammation, causing an inflammatory condition that stimulates genetic instability, cell proliferation, and metastatic capability participating to cancer progression [29].

As far as MM is concerned, the role played in this pathology by the NF-kb pathway is well known [30].

In fact, the activity of AGE/RAGE ligand in the mitogen-activated protein kinase (MAPK)/NF-κB cascade signaling pathway is of particular interest [31]. The activation of a signal transducer and activator of transcription factor 3 (STAT3), stimulated by several mechanisms comprising RAGE-dependent pathways, triggers extended initiation of NF-kB [32]. NF-kB and STAT3 signaling pathways provoke tumor-stimulating reactions by the production of interleukin-6 (a well-known growth factor for myelomatous plasma cells), prostaglandin E2, matrix metalloproteinases, and RAGE stimulation in the microenvironment of a tumor [33,34,35].

**Figure 4:**
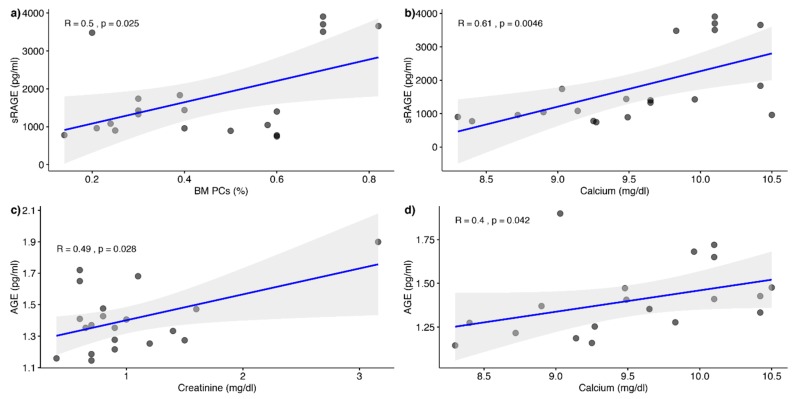
Correlation between AGE/RAGE vs significant blood parameters.

In our study, AGE levels were significantly reduced in patients with respect to controls, while sRAGE concentrations were significantly elevated in patients affected by MM compared to healthy subjects. Moreover, we found a correlation between the levels of AGE and two of the parameters used to evaluate organ damage, such as creatinine and calcium, and a correlation between sRAGE and calcium (Figure 4).

Although AGEs and AOPPs are usually markers of a pro-inflammatory or of an oxidative and pathological status, the data obtained seem to controvert the literature. However, this is not the first time that similar data were described, although in other diseases [36]. Some researchers speculated that AGE-modification renders macromolecules susceptible for elimination via the scavenger receptor of endothelial and Kupffer cells [37].

It is well known that AGEs are implicated in the pathogenesis of bone-destructive disorders. AGEs stored in the bone matrix have the ability to reduce osteogenic and increase osteoclastogenic properties of osteoblasts in vivo, leading to structural damage of bone [38,39].

RAGE is also involved in bone remodeling [40], and the cross-linking in the bone matrix by AGEs can influence the fracture resistance of bone [41].

Engblom et al. showed that sRAGE was increased in the serum of lung tumor-bearing mice and that sRAGE accounts for the osteoblast activation [42]. During the last decade, it was reported that AGE is not the only ligand of soluble RAGE; also, AOPP and other pro-inflammatory mediators could bind to the soluble receptor. Therefore, its higher levels in MM patients could confirm the pathological status [43,44]. The highest rates of sRAGE were observed in IgA lambda MM patients compared to IgA kappa, IgG kappa, and IgG Lambda groups. In this regard, numerous studies have highlighted the possible prognostic significance of the type of light and heavy chains in MM patients. In an older study, it was found that the median survival of patients with IgA/lambda paraproteinemia was apparently shorter than that of other subgroups [45], while in a different stuy it was demonstrated that patients who had two different lambda light chain have a poor prognosis [46]. Other authors described the possibility of administering sRAGE in order to counteract the detrimental effects of AGEs exposition in an anti-oncogenic sense [47]. In accordance with this speculation, Galliera et al. conducted a study in patients affected by metastatic cancers, concluding that sRAGE could play a protective role in bone metastasis progression, justifying a reactive augmentation in MM subjects [48].

In our MM patients, we also found an increase in sRAGE levels correlating with the number of bone marrow plasma cells. Furthermore, in multivariate analysis, the calcemia and beta2 microglobulin values and the number of bone marrow plasma cells were predictive of sRAGE concentration.

Our study demonstrated that the role of the sRAGE/AGE axis in patients with MM, although of promising importance, has still to be better clarified. It is clear, however, that sRAGE is the prevailing form of the receptor counteracting RAGE-mediated pathogenesis by operating as a snare receptor [49,50]. Therefore, it has been proposed as an endogenous factor against pathogenesis eased by ligands-RAGE axis, including tumor development [51]. The sRAGE pool is not able to entirely exclude the circulating AGE in vivo. Since AGE is a positive regulator of the activity of RAGE, circulating sRAGE concentrations may reflect RAGE expression and may be increased in parallel with serum AGE concentrations as a counter-system against AGE-caused tissue damage [52,53].

From this point of view, serum concentrations of AGE and sRAGE could therefore not only be useful markers in MM patients, but they could also become potential therapeutic targets.

The action of RAGE and its ligands may be reduced in several possible ways using both siRNAs, monoclonal antibodies, or small molecules [54].

In conclusion, data obtained by in vitro and in vivo reports proved how RAGE inhibition could be a useful way for the management of numerous diseases. Independent of the model employed, the reduction of RAGE provoked a normalized inflammatory response as demonstrated by the decrease in NF-κB activity, cytokine generation, and RAGE expression [55].

The changeable nature of AGEs and sRAGE theoretically will offer a chance for MM treatment if their actions in its development are clarified.

Though our study had a small number of participants, there appeared to be relevant modifications in the sRAGE/AGE axis of MM patients and controls, indicative of a promising role in MM pathogenesis. Further studies should better assess this role by evaluating more inflammatory markers and pro- and anti-tumorigenic mediators.

## Figures and Tables

**Table 1:** Correlation between variables.

Values and Parameters	sRAGE	AGE	age	MC	BM PCs	CREATIN.	Beta2Microg.	Calcemia	Hb
sRAGE	1								
AGE	0.37	1							
age (yrs)	0.15	0.0062	1						
MC	0.23	−0.15	0.0061	1					
BM PCs_	0.50 *	0.19	−0.29	0.48 *	1				
CREATININE	−0.081	0.49 *	0.17	0.15	−0.04	1			
Beta2Microg.	0.33	−0.022	0.46 *	0.26	−0.29	0.29	1		
Calcemia	0.61 ***	0.40 *	0.12	−0.19	0.23	−0.21	0.096	1	
Hb	−0.37	0.17	−0.53 *	−0.058 ***	−0.31	−0.052	−0.49 *	−0.18	1

sRAGE = soluble receptor for advanced glycation end-products; AGE = advanced glycation end-products; age = patients age; MC = monoclonal component; BM PCs = bone marrow plasma cells; creatin. = serum creatinine; beta2microg. = beta 2 microglobulin; calcemia = calcemia; Hb = hemoglobin.

**Table 2:** Microglobulin, bone marrow plasma cells, and the interaction between calcemia and bone marrow plasma cells were the best combination of model variables which significantly predicted sRAGE.

Model/Variables Using Variables Most Highly Correlated with sRAGE	Adjusted R-square
BM PCs + Calc	0.447
BM PCs + Beta2Microglob. + Calc	0.613
BM PCs + Beta2 Microglob. + Calc: PCs	0.746

## Supplementary Materials

The following are available online at https://www.mdpi.com/2076-3921/8/3/55/s1, Table S1: Table describing patients age and sex (7 M–12 F; mean age 71.63+/−9.96), Table S2: Table reporting AGE fluorescence values (patients average: 1362 pg/ml +/−0,186; controls average 1517+/−0.152), Table S3: Average age values for male and female patients.
