# Supplementary material for: Evaluation of the AGE/sRAGE Axis in Patients with Multiple Myeloma
Source: Antioxidants (Basel). 2019 Mar 4;8(3):55. doi: 10.3390/antiox8030055 (PMC6466542; doi:10.3390/antiox8030055)
Supplement: Supplementary file 1 [file antioxidants-08-00055-s001.pdf]

antioxidants

MDPI

Supplemental Material:

S= supplemental

Table S1. Table describing patients age and sex (7 M – 12 F; mean age 71.63+/-9.96).

Sex	Age
F	67
M	77
M	57
F	86
F	84
M	63
M	60
M	73
F	75
F	76
F	69
F	87
F	55
M	65
F	89
M	74
F	70
F	66
F	68

Table S2. Table reporting AGE fluorescence values (patients average: 1,362 pg/ml +/-0,186; controls average 1,517 +/-0,152).

Patients			MEAN	10^-6 (pg/ml)
1	1.404.000	1.415.000	1409500	1,4095
2	1.404.000	1.301.000	1352500	1,3525
3	1.891.000	1.907.000	1899000	1,899
4	1.291.000	1.263.000	1277000	1,277
5	1.304.000	1.244.000	1274000	1,274
6	1.296.000	1.209.000	1252500	1,2525
7	1.302.000	1.438.000	1370000	1,37
8	1.664.000	1.698.000	1681000	1,681
9	1.185.000	1.132.000	1158500	1,1585

10	1.168.000	1.212.000	1190000	1,19
11	1.467.000	1.485.000	1476000	1,476
12	1.157.000	1.132.000	1144500	1,1445
13	1.340.000	1.325.000	1332500	1,3325
14	1.348.000	1.358.000	1353000	1,353
15	1.454.000	1.357.000	1405500	1,4055
16	1.484.000	1.459.000	1471500	1,4715
17	1.454.000	1.400.000	1427000	1,427
18	1.226.000	1.205.000	1215500	1,2155
19	1.168.000	1.212.000	1190000	1,19
Controls			MEAN	10^-6 (pg/ml)
1	1.302.000	1.316.000	1309000	1,309
2	1.458.000	1.553.000	1505500	1,5055
3	1.436.000	1.442.000	1439000	1,439
4	1.366.000	1.400.000	1383000	1,383
5	1.625.000	1.730.000	1677500	1,6775
6	1.459.000	1.435.000	1447000	1,447
7	1.320.000	1.281.000	1300500	1,3005
8	1.282.000	1.287.000	1284500	1,2845
9	1.449.000	1.503.000	1476000	1,476
10	1.669.000	1.844.000	1756500	1,7565
11	1.592.000	1.661.000	1626500	1,6265
12	1.809.000	1.730.000	1769500	1,7695
13	1.545.000	1.672.000	1608500	1,6085
14	1.514.000	1.606.000	1560000	1,56
15	1.562.000	1.546.000	1554000	1,554
16	1.620.000	1.523.000	1571500	1,5715

Table S3. Average age values for male and female patients.

Gender	Number patients	Average Age	Fluorescence
Male	7 patients	67 +/- 7.7	1482785 +/- 228115
Female	12 patiens	74.3 +/- 10.4	1291666+/- 115678
